# Implementing oral (event-driven and daily) and long-acting pre-exposure prophylaxis in mobile men in sub-Saharan Africa: a phase 3b, open-label, hybrid type 2 implementation and effectiveness trial (MOBILE MEN)

**DOI:** 10.1186/s13063-025-09138-5

**Published:** 2025-11-10

**Authors:** Sylvia Kusemererwa, Bernadette Nayiga, Limakatso Lebina, Linda Gail Bekker, Andrew Philips, Loveleen Bansi- Matharu, Darshini Govindasamy, Gesine Meyer-Rath, Geofrey Kimbugwe, Eugene Ruzagira, Maryam Shahmanesh, Janet Seeley, Emily L. Webb, Julie Fox

**Affiliations:** 1 Medical Research Council/Uganda, Virus Research Institute and London School of Hygiene and Tropical Medicine Uganda Research Unit , Entebbe, Uganda; 2 Africa Research Health Institute, Durban, South Africa; 3 Desmond Tutu HIV Foundation, Cape Town, South Africa; 4 University College London, London, UK; 5 South African Medical Research Council, Cape Town, South Africa; 6 University of the Witwatersrand, Johannesburg, South Africa; 7 London School of Hygiene and Tropical Medicine, London, UK; 8 King’s College London, London, UK

**Keywords:** HIV, PrEP, Long-acting cabotegravir, Mobile populations, Sub-Saharan Africa, Men

## Abstract

**Background:**

Men who are mobile for work are a key population at high risk of acquiring HIV. Flexible pre-exposure prophylaxis (PrEP) options, including event-driven (ED) oral PrEP and long-acting injectable cabotegravir (CAB-LA), may offer increased access and acceptability for these men. However, limited data exist on the effectiveness and implementation of CAB-LA and ED PrEP among mobile men in Africa. Our study aims to assess the effectiveness and implementation of CAB-LA and oral tenofovir disoproxil fumarate/emtricitabine (TDF/FTC) (both daily and ED) through comparison of uptake, retention in care, coital coverage, and participant choice.

**Methods:**

We will conduct a mixed0method, phase 3b, open-label, hybrid type 2 implementation and effectiveness randomised controlled trial (RCT). The trial will be carried out in 400 HIV-negative men aged 18 years or older in South Africa and Uganda. Men will be randomised 1:1 to either Group A: oral TDF/FTC PrEP (ED or daily) or Group B: CAB-LA over 9 months. After 9 months, participants from both groups will be offered a choice of PrEP (oral TDF/FTC or CAB-LA) for a further 9 months, with the ability to change their choice as required. Various strategies to support PrEP adoption, initiation, and persistence will be implemented, monitored, and reported on using a RE-AIM (reach, effectiveness, adoption, implementation, and maintenance) implementation science framework.

**Discussion:**

This study will provide critical data to inform scalable delivery models for both oral and injectable PrEP among mobile men at high risk for HIV acquisition. Findings will also highlight the potential of PrEP choice delivery and its benefits, offering evidence for governments to consider in the rollout of injectable PrEP in public health systems.

Trial registration

NCT06133686. Registered on 14 November 2023. PACTR202409632006463. Registered on 2 September 2024.

## Introduction

### Background and rationale {6a}

Whilst daily HIV prevention pre-exposure prophylaxis (PrEP) has been extensively evaluated in men who have sex with men and adolescent girls and young women (AGYW) [1], there have been few evaluations of injectable and event-driven (ED) PrEP in men who mainly have sex with women in sub-Saharan Africa. ED PrEP is a regimen that involves taking two pills 2–24 h before sex and then one pill every 24 h until 48 h after the last sexual encounter [2]. Research to fill this gap is required to improve PrEP guidelines and facilitate successful PrEP rollout, a key focus of the Sustainable Development Goal target to end AIDS by 2030 [3].

ED PrEP for men, regardless of sexual orientation or sexual modality, has been recommended by WHO since 2019 [4], but this approach has not been endorsed in South Africa and only recently endorsed in Uganda [5] despite evidence of high acceptability demonstrated in feasibility studies [6]. Compared to daily oral PrEP, ED PrEP may require fewer tablets, which will lead to lower costs and fewer side effects. More recently, injectable PrEP has been shown to be more effective than daily oral PrEP [7] mainly because of better adherence, and as a result, long-acting cabotegravir (CAB-LA) has been recommended for use since 2022 [8].

To support rollout of CAB-LA where the HIV burden is greatest, there is a need for studies to include men from sub-Saharan Africa. Acceptability studies have indicated that men show a preference for CAB-LA over oral PrEP; however, this is yet to be trialed [8–12]. Furthermore, there is a need to develop affordable and accessible HIV testing strategies for CAB-LA monitoring so that it can be delivered safely in primary care settings. Although the WHO recommends the use of national HIV testing algorithms for those on CAB-LA, this approach is more likely to miss early infection than nucleic acid-based tests, particularly at CAB-LA initiation [13]. Conversely, HIV viral load testing in CAB-LA is problematic as such tests lack regulatory approval; HIV diagnostic tests are expensive and require laboratories. Furthermore, clinical trials of CAB-LA have found positive tests very difficult to interpret [14, 15]. HIV self-tests have not been used in CAB-LA provision so far, and their use to increase the frequency of HIV testing may increase detection of early HIV infection.

An important factor in successfully enabling people to protect themselves is choice. Oral and injectable PrEP offer different benefits. The short-acting oral agents offer the ability to discontinue PrEP rapidly when desired. While long-acting PrEP removes the need for tablets and potentially improves adherence, it increases the number of clinic visits and the need for a trained health care worker to give the injection [16]. With the introduction of CAB-LA, a concern amongst providers is that it could lead to the re-medicalization of PrEP, hindering the migration of PrEP services into community venues (outside of clinic facilities) and the administration of PrEP by lay health workers [4, 17]. The delivery of injectable PrEP in community settings and for mobile groups is essential for widespread rollout.

Little is known about the most effective way to implement PrEP choice for men in Uganda and South Africa. Engaging men and retaining them in HIV prevention and treatment programmes have been met with challenges in sub-Saharan Africa [18]. Men consistently fare worse than women in levels of HIV testing and ART initiation [18, 19]. Indeed, the failure of some Treatment As Prevention (TASP) trials to show effect was mainly due to the inability to test and treat young men [20]. Providing PrEP for men therefore represents a large unaddressed gap in HIV services in Africa [21]. In South Africa and Uganda, other than voluntary medical male circumcision and condom promotion, there have been few prevention options for heterosexual men. Men have had limited access to oral PrEP in part due to poor access to primary health care services, due to stigma, inconvenient opening times and long waiting times, and due to a lack of knowledge of HIV prevention options [21–23].

Men who are mobile due to work or looking for work are a key population at high risk of acquiring HIV [21]. High HIV prevalence remains disproportionately high in this population in sub-Saharan Africa. Estimates range from 0.95% to 54% among truck drivers [24], 39.5% to 42% among farm workers [25], 26% among unemployed men in South Africa [26], and 40% among men in fishing communities in Uganda [27]. As Africa’s large youth bulge moves into young adulthood, the search for work and associated mobility will further increase. Despite this, PrEP intervention studies have not included these populations [28], and, as such, a real-world delivery model for HIV prevention in mobile men has not been developed. One specific concern is that those who are mobile for work may require more flexible PrEP delivery systems to allow for extended periods away from home [4].

Political decisions regarding rollout and delivery models need to take into account implementation needs and cost-effectiveness. Recent economic analyses comparing CAB-LA to oral PrEP have shown mixed results. Seven studies modelled data and scenarios specific to South Africa [29–35], but with the exception of two [35, 36], none was based on trial or implementation study data. Additionally, one study assessed costs for men who have sex with men and transgender women in the USA [37]. Economic analyses comparing CAB-LA to oral PrEP based on data from implementation and countries other than South Africa are therefore needed. The Mobile Men study will provide data on the cost and outcomes of ED PrEP and HIV antibody test monitoring of CAB-LA to be used for such economic analyses. We also, for the first time, will provide cost data for Uganda for oral PrEP and CAB-LA PrEP. For this, we will use the HIV Synthesis model, an individual-based model that includes both oral and long-acting injectable PrEP, while accounting for drug resistance [36, 38]. This is particularly important for CAB-LA, given its tail dosing and the potential for cross-resistance between cabotegravir and dolutegravir, the cornerstone drug of first-line HIV treatment globally.

To improve uptake, persistence, and effective use of all forms of PrEP in men, there is a need for simplified and differentiated delivery of PrEP that is person- and community-centred. Effective use requires that all potentially high-risk sex acts be covered by an effective preventive intervention such as PrEP. Given the importance of flexibility due to travel in this group, our hypothesis is that both oral PrEP (in particular, ED PrEP) and CAB-LA PrEP will be highly acceptable to men in two high-burden, resource-limited African countries, with high levels of effective use including persistence and coital coverage. Policy makers will need evidence not only around effective use but also the reach, adoption and choice, implementation and maintenance of PrEP in different settings, and with different high-risk mobile men to inform generalisability and scalability of PrEP for heterosexual men.

The Mobile Men study is the first PrEP study implementing both ED oral PrEP and CAB-LA targeting mobile men in sub-Saharan Africa (South Africa and Uganda). The study will provide the evidence for effectiveness, implementation, and cost-effectiveness, which are critical to decision-making by African governments and donors on how to prioritise prevention resources and inform guidelines.

## Objectives {7}

The overall objective of the Mobile Men study is to assess the effectiveness and implementation of CAB-LA and oral TDF/FTC (both daily and ED) amongst men who are mobile for work in South Africa and Uganda, through comparison of uptake, retention in care, coital coverage, and participant choice.

The primary user effectiveness objective is to compare short-term (9 months) and longer-term (18 months) PrEP persistence patterns across different PrEP modalities (oral and injectable) amongst men who are mobile for work in South Africa and Uganda.

The primary implementation objective is to compare adoption (uptake and choice) across the different PrEP modalities (oral and injectable) amongst men who are mobile for work in South Africa and Uganda.

The secondary clinical objectives are as follows:

- To compare the effective coital coverage of oral daily/ED TDF/FTC versus CAB-LA
- To describe the safety, tolerability, and acceptance of all methods, as determined by self-reported side effects, adverse events (AEs), and reasons for PrEP pause or discontinuation

The secondary implementation objectives are as follows:

- To understand the reach of PrEP for mobile men and understand the barriers to, and facilitators of, uptake amongst those at risk who do and do not accept oral or injectable PrEP
- To describe adoption
- To identify characteristics of men who adopt oral PrEP versus CAB-LA
- To describe patterns of use of daily, ED, and long-acting PrEP amongst different groups of mobile men
- To understand the Implementation of ED and long-acting PrEP for mobile men amongst service providers to inform scale-up
- To understand the feasibility and fidelity of delivering ED and long-acting PrEP in different settings
- To describe how ED and long-acting PrEP are delivered in practice
- To describe the service-level needs to implement ED and long-acting PrEP
- To evaluate acceptability and implementation of antibody-based HIV status monitoring for CAB-LA
- To assess the total and average cost of oral PrEP and CAB-LA in South Africa and Uganda under study conditions from the provider perspective (maintenance)

The exploratory objectives are as follows:

1. To describe HIV incidence across the two PrEP modality arms
2. To describe any evidence of HIV drug resistance mutations in participants who acquire HIV infection whilst using PrEP across the two PrEP modality arms
3. To describe persistence in care of those diagnosed with HIV at screening
4. To describe body mass index (BMI) and blood pressure across both arms

## Trial design {8}

The Mobile Men study is a phase 3b, open-label, hybrid effectiveness-implementation type 2 trial to evaluate the initial implementation (scale-up) phase of CAB-LA as a PrEP option in men who are mobile for work in South Africa and Uganda with a high burden of HIV. The trial is a phase 3b, open-label, hybrid type 2 study with co-primary aims of effective use and implementation. The implementation of PrEP choice in mobile men is further investigated using a mixture of social science and health economics methods to understand reach, adoption, implementation, and maintenance. These include repeat rapid ethnographic assessments [39], qualitative interviews with users and providers, process evaluation, modelling and cost-effectiveness analysis.

**Fig. 1:**
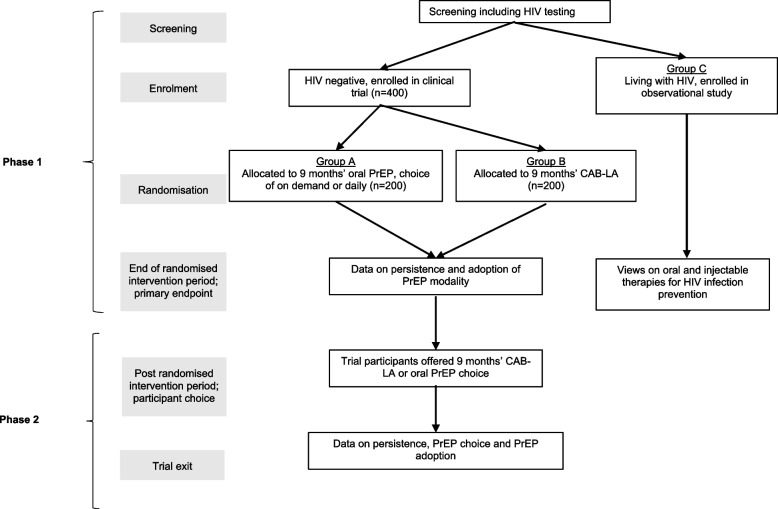
Schematic of study design

A schematic representation of the trial design is shown in Fig. 1. Participants will be screened for HIV at enrolment. Those testing negative will be randomised to receive either oral or injectable PrEP for an initial 9-month period. Then, throughout the 9- to 18-month period following randomisation, participants will be offered a choice of oral or injectable PrEP. Participants determined as living with HIV at screening will be linked to care and followed up as a separate cohort alongside randomised trial participants.

An embedded social science component provides data on the process of trial implementation as well as detailed information on study sites, local communities, and perspectives on both trial and trial products by participants. This component of the study is divided into three parts: a rapid ethnographic assessment conducted at three time points during the trial period (before, during, and at the end); qualitative data collection with trial and non-trial participants and trial staff throughout the trial; using in-depth interviews, group discussions, observations, and structured debriefings; and a health economics component addressing costs and cost-effectiveness.

In this hybrid effectiveness-implementation type 2 trial, effectiveness outcomes will be assessed within a superiority hypothesis testing framework, and implementation outcomes within an exploratory framework, in order to generate evidence to inform future scale-up of HIV PrEP by identifying which product modalities and delivery strategies are most acceptable, feasible, and effective for mobile men in resource-limited, high HIV burden settings.

## Methods: participants, interventions, and outcomes of the trial

### Study setting {9}

The study will take place at three sites: two in South Africa [KwaZulu-Natal and Buffalo City Metro (BCM) Municipality, Eastern Cape] and one in Uganda (Masaka city, Masaka district). In South Africa, the study will be conducted by the Africa Health Research Institute in KwaZulu-Natal and the Desmond Tutu Health Foundation in East London. In Uganda, the study will be conducted by the Medical Research Council/Uganda Virus Research Institute and London School of Hygiene and Tropical Medicine Uganda Research Unit.

### Eligibility criteria for the trial {10}

The inclusion criteria are as follows:

- Ability and willingness to provide informed consent
- Age 18 years or older
- Willingness to undergo HIV testing
- Assigned male at birth
- History of work-related travel in the past 6 months, including spending at least one night away from home for work purposes and being at risk of HIV infection
- Willingness to use PrEP

The exclusion criteria are as follows:

- Confirmed HIV infection
- Reasons at the discretion of site investigator for unsuitability for study inclusion
- Body weight less than 35 kg
- Use of contraindicated medications including the following: Medication for tuberculosis (e.g. rifampin, rifapentine) and anticonvulsants (e.g. carbamazepine, oxcarbazepine, phenobarbital, phenytoin)
- Known allergy to any of the study products

### Who will take informed consent? {26a}

Informed consent will be obtained by trained study staff delegated by the principal investigator. At the screening visit, participants will have the opportunity to ask questions and have a full discussion of the information provided in writing and/or visually and verbally. This will include the following key messages:

- That there is a 1 in 2 chance that the participant will be assigned to CAB-LA and a 1 in 2 chance that they will be assigned to oral PrEP
- That condoms and PrEP are known to reduce the risk of acquiring HIV when used or taken consistently

Participants will be shown images of the PrEP pills and the CAB-LA vials. They will be asked if they discussed their intention to participate in the study with anyone and if any problems arose because of this. If they wish to proceed, they will be given an informed consent form to read and sign prior to any study procedures. The informed consent form will be dated and countersigned by the investigator or delegated person, who administers the informed consent process. If the participant is unable to read and write, they will be asked to place their thumbprint on the informed consent form in the presence of an independent witness who will have been present during the discussion. The witness cannot be a member of the site study staff. The right of the participant to refuse to participate without giving reasons will be respected.

A copy of the consent form will be provided to the participant,and one copy was kept securely in the study file according to local procedures. For the qualitative component of the study, the participant information sheet for the in-depth interviews and group discussions will be used to provide information to study participants in one-on-one conversations. Written informed consent will be obtained prior to conducting interviews by staff trained in interview techniques.

### Additional consent provisions for collection and use of participant data and biological specimens {26b}

Additional consent will be obtained to collect and store blood samples from people who acquire HIV during the study.

## Interventions

### Explanation for the choice of comparators {6b}

Daily oral PrEP is effective, but a substantial proportion of people offered this approach either fail to initiate it, discontinue relatively soon after starting, or do not use it effectively [18]. While the WHO endorses ED PrEP for men, regardless of sexual orientation or sexual modality, this regimen has not been taken up in South Africa and has only recently been introduced in Uganda [5]. To date, no efficacy studies have been carried out using ED PrEP for heterosexual men in Africa. Research carried out by our consortium recently showed that ED PrEP is the oral prevention method of choice among young men in South Africa, Uganda, and Zimbabwe [6]. The advantage of oral ED PrEP over daily PrEP means that potentially fewer tablets are required which lowers the cost, minimises side effects, and potentially lowers the risk of stigma due to reduced visibility of PrEP use, particularly when travelling for work.

CAB-LA is a strand transfer integrase inhibitor that is delivered as a suspension via a gluteal intramuscular injection every 2 months. Two double-blind, double-placebo studies, HPTN 083 and HPTN 084, have shown CAB-LA is superior to daily oral TDF/FTC, with significantly lower HIV incidence observed in the CAB-LA arms across both male and female populations [7, 13]. HPTN 083 was conducted amongst cis-gender men and transgender women who have sex with men in the USA, Latin America, Asia, and Africa. Participants in the CAB-LA arm had a 66% lower risk of HIV infection compared to their counterparts in the TDF/FTC arm [13]. HPTN 084 was conducted amongst cis-gender women across seven sub-Saharan African countries. Participants in the CAB-LA arm had an 88% lower risk of HIV infection compared to those in the TDF/FTC arm [7].

### Intervention description {11a}

Eligible participants (men testing HIV negative at screening) will be randomised in a 1:1 ratio to either Group A: oral TDF/FTC PrEP (ED or daily) or Group B: CAB-LA over 9 months. After 9 months, participants from both groups will be offered a choice of PrEP (oral TDF/FTC or CAB-LA) for a further 9 months, with the ability to change their choice as required.

### Criteria for discontinuing or modifying allocated interventions {11b}

There are no planned modifications to oral TDF/FTC or CAB-LA PrEP dose. PrEP must be interrupted if a participant suffers a serious adverse event (SAE) that could be a drug reaction. Participants who have a positive hepatitis B virus test will be recalled if they are using CAB-LA PrEP and invited to switch to oral PrEP at the end of month 1 or counselled on alternative HIV prevention products.

If an individual chooses to stop PrEP, they may remain in the study, and the reason for stopping is documented.

The study investigator can also discontinue an individual from their allocated intervention for the following reasons:

- Confirmed HIV infection
- Unacceptable toxicity that precludes the continuation of PrEP
- Intercurrent illness that prevents the safe use of PrEP
- Early termination of study

Participants who enrol on the study and/or have received at least one CAB-LA injection or one PrEP tablet will be asked to remain in follow-up. However, participants may withdraw their consent for further visits at any point during the trial. Data that are already collected from participants who stop follow-up early may still be analysed, provided they have given consent.

### Strategies to improve adherence to interventions {11c}

Participants will be offered counselling to enhance PrEP adherence and attend scheduled study appointments. At enrolment, participants will be asked for a primary and secondary phone number, if they have one, as well as their home address. They will also be asked whether they consent to home visits by the study team in case of missed study visits. Additionally, a clinical hotline will be provided to enable participants to directly contact the study team for assistance.

### Oral PrEP

All participants will receive detailed information on how to correctly use PrEP. Adherence to oral PrEP will be assessed using self-reported data, pill counts of dispensed medication, and drug concentration levels measured in dried blood spots (DBS).

### Injectable PrEP

All injections will be administered by site staff and recorded in the relevant case report form (CRF). If an injection is not given within the ideal window, the deviation and reason for it will be recorded in the CRF.

### Relevant concomitant care permitted or prohibited during the trial {11d}

No concomitant medications are prohibited for TDF/FTC. Concomitant medications that are prohibited for CAB-LA are rifampicin, carbamazepine, oxcarbazepine, phenytoin, phenobarbital, and rifapentine.

### Provisions for posttrial care {30}

All participants exiting the trial will be signposted and linked to HIV prevention services that provide PrEP. It may be that locally available CAB-LA is not yet in place, and this will be discussed with participants wanting to access it.

### Outcomes {12}

The trial will have two co-primary outcomes:

- User effectiveness co-primary outcome: This will assess persistent use of PrEP during the randomised period and throughout the full follow-up period. Persistent use of PrEP during the randomised period will be defined as a binary outcome. Participants randomised to the CAB-LA arm who receive all injections as per schedule (initial injection at month 0, followed by injections at months 1, 3, 5, and 7, with an acceptable window of ±7 days for each injection) will be considered “persistent on PrEP”. Participants who do not receive all injections as scheduled will be classified as “non-persistent on PrEP”, regardless of the frequency or timing of self-reported condomless sex. Participants randomised to the oral PrEP arm will be considered “persistent on PrEP” if they attend scheduled 3-monthly visits, receive sufficient PrEP to maintain supply until the next visit, and have detectable TFV-DP levels in DBS.
  During the full follow-up period (18 months), persistence will be assessed as a multinomial outcome with the following categories: single modality, consistent use; multiple modalities, consistent use; single or multiple modalities, inconsistent use; and never started PrEP.
- Implementation co-primary outcome: This will compare the adoption (uptake when offered and choice preference of the different modalities). Uptake is defined as the proportion of participants who are offered and subsequently take PrEP (oral PrEP arm versus CAB-LA arm) during the 9-month randomised period. Choice preference will be measured as the proportion of participants who choose and use each modality (daily or ED oral PrEP or CAB-LA), captured at the start and the end of the subsequent 9-month choice period.

Secondary outcomes include the following:

- Short-term flexible PrEP persistence: A binary outcome that is analogous to the user effectiveness co-primary outcome but with more flexibility allowed on visit windows, specifically an acceptable window of −7 to + 28 days for scheduled injections at months 3, 5, and 7 in the CAB-LA arm and for scheduled visits at months 4 and 7 in the oral PrEP arm.
- Effective peri-coital coverage of PrEP: A binary outcome measuring whether, at the time of last condomless sex, the participant was “covered” by PrEP
- Safety, tolerability, and acceptance: Assessed based on self-reported AEs and the reasons for PrEP pause or discontinuation
- Reach: Evaluated qualitatively through three serial rapid ethnographic assessments capturing information on awareness and reach of PrEP amongst mobile men
- Adoption: This will be evaluated by (i) comparing the characteristics of men who uptake oral PrEP with those who uptake CAB-LA when offered at month 0, (ii) comparing the characteristics of men who choose oral PrEP with those who uptake CAB-LA when given the choice at month 9, and (iii) describing patterns of use of daily, ED, and long-acting PrEP during months 9–18 of the trial and the transitions between these modalities.
- Implementation: Evaluated using qualitative data collected from service providers, with a focus on fidelity and feasibility
- Maintenance: Evaluated through calculation of total and average cost of oral PrEP and CAB-LA in South Africa and Uganda under study conditions, from the provider perspective

### Participant timeline {13}

The participant timeline is shown in Table 1.

**Table 1:** Mobile Men study schedule of enrolment, interventions, and assessments

	**Enrolment**	**Allocation**	**Randomisation phase**						**Choice phase**		**Close-out**
**Timepoint**	**0**	**0**	**M0**	**M1**	**M3**	**M4**	**M5**	**M7**	**M9**	**M10–17**^**1**^	**M18**
**Enrolment**											
Eligibility screen	X										
Informed consent	X										
Allocation		X									
**Intervention**											
CAB-LA injection			X	X	X		X	X			
Oral PrEP dispensed			X	X		X		X			
**Assessments**^**2**^											
Locator information	X			X		X			X	X	
Demographics	X										
Risk and mobility (short CRF)	X			X	X	X	X	X	X	X	
Risk and mobility (Long CRF)	X								X		X
PrEP adherence				X	X	X	X	X	X	X	X
Adverse events			X	X	X	X	X	X	X	X	X
IDIs				X		X	X		X	X	X
FGDs				X					X		X
Surveys	X								X		X
HIV test	X			X	X	X	X	X	X	X	X
STI screen	X								X		
DBS	X			X	X	X	X	X	X	X	X
Blood pressure	X				X	X			X	X	X
BMI	X				X	X			X	X	X
CrCl if ≥ 30 years	X										
HepBsAg	X										

^1^Visit schedule for choice phase is dependent on choice of PrEP made at month 9 and at each subsequent visit. ^2^Done at visits corresponding to the intervention received by the participant; *CAB-LA*, long-acting injectable cabotegravir; *PrEP*, preexposoure prophylaxis; *CRF*, case report form; *IDIs*, Iin-depth interviews; *FGDs*, focus-group discussions; *HIV*, human immunodeficiency virus; *STI*, sexually transmitted infection; *DBS*, dried blood spot; *BMI*, body mass index; *CrCl*, creatinine clearance; *HepBsAg*, hepatitis B surface antigen

### Sample size {14}

The planned enrolment sample size of 400 participants (randomised) will allow 80% power to detect an absolute difference of 11% in persistence between trial arms at a 5% significance level, assuming 80% persistence in the lower persistence arm [40] (and 91% persistence in the higher persistence arm) and allowing for 20% lack of ascertainment of persistence, for example, due to medical ineligibility to receive the randomised intervention or death. If the persistence in the lower persistence arm is lower, at 60% [41], then we will have 80% power to detect an absolute difference of 15% (i.e. 60% versus 75% persistence). Sample size is calculated using standard methods for comparing two proportions and implemented using the “power two proportions” command in STATA.

### Recruitment {15}

#### Preparatory work

Preliminary work in the communities is being carried out, including a rapid ethnographic assessment study to inform and facilitate study implementation. Demand creation for PrEP will be achieved through a community engagement campaign and peer and sex worker outreach activities prior to and during the study start. These efforts will expose men in the communities to PrEP educational materials and encourage them to visit community and public health sites to access sexual health services, including PrEP. Potential participants will be provided with educational information about each of the PrEP products both during the enrollment visit and as part of the broader community-wide demand creation campaign preceding the study.

### Masaka, Uganda

Recruitment efforts will mainly focus on landing sites around the shores of Lake Victoria in Masaka District. These fishing communities have a high HIV burden that is attributed to several factors, including high mobility, sex work, relatively young populations with disposable daily income, and inadequate health services. People in these communities frequently move between different landing sites and islands, with up to 47% of men and 25% of women reporting being away from home for at least 2 days each month. This mobility is mainly driven by the seasonal fish catch. Previous studies have highlighted that such high mobility can limit access to health services, increase HIV infection risk, and underscore the need for tailored HIV prevention interventions that account for these mobility patterns [39, 42]. The Mobile Men study has been designed to address these challenges.

The study team will work with local leaders and members of Village Health Teams and via community HIV testing services to identify potential participants from places of entertainment (such as lodges/guest houses, bars) and from their homes. Detailed locator information, including addresses, telephone contacts, and next-of-kin details, will be collected to facilitate effective phone and/or physical tracing during the follow-up phase of the study.

### Buffalo City Metro (BCM) Municipality, Eastern Cape, South Africa

The Eastern Cape province is a historically under-resourced and under-researched province. Since 1990, Eastern Cape has ranked last among South Africa’s nine provinces in its Human Development Index score [43, 44]. In 2022, the estimated HIV prevalence in the province was 19.4% among individuals aged 15 to 49 years. That same year, an estimated 92.6% of individuals living with HIV in Eastern Cape knew their status, 74.9% were on ART, but only 62.6% were virally suppressed (Viral load < 1000 copies/mL) [45, 46]. Given that South African men are less likely to know their HIV status, initiate ART, or be virally suppressed compared to women, we expect these indicators will be worse for men in the province [47].

Within BCM, men will be recruited from Duncan Village (urban) and Mdantsane (peri-urban) townships. These predominantly black, Xhosa-speaking communities have some of the highest HIV prevalence rates in South Africa. Both Duncan Village and Mdantsane are densely populated, with many residents living in informal housing. Mdantsane, in particular, is one of the largest townships in the country. Mobile men from these communities return home on a regular basis to visit their extended families. Recruitment will target men who sit by the roadside waiting for offers of casual labour. Previous studies have reported an HIV prevalence of 16.6% in this population [48]. Additionally, construction workers and migrant workers (men who leave their partners and families to work) will be recruited. Truck drivers will also be targeted through nurse-led primary healthcare services at a fixed roadside Wellness Centre located near East London’s shipping port and airport. The centre serves an extremely busy route for the long-distance goods transport.

### KwaZulu-Natal, South Africa

Recruitment will focus on mobile young men from a busy urban and peri-urban taxi rank and market area in Mtubatuba town in uMkhanyakude where the main N2 highway intersects with the R618 route to St. Lucia and surrounding areas. This primarily rural area faces significant economic challenges compared to other regions in South Africa, with high levels of unemployment. For example, over 85% of young people aged 20–24 are unemployed. The uMkhanyakude district also has a very high HIV prevalence of 30% [49]. Healthcare infrastructure is limited, with only 1 public hospital and 13 fixed-location primary healthcare clinics, all of which face resource constraints in delivering HIV prevention and health promotion [50]. Men looking for work, or on their way to find work in the larger urban setting of Durban and Johannesburg, the Richards Bay shipping port, the coalmines, and sugar plantations of uMkhanyakude, will mostly transition through the taxi rank and market area of Mtubatuba. It is also a hub where men who move for work spend their earnings on food, sex work/transactional sex, other recreational activities, and alcohol. Stakeholder engagement, key informant interviews, and rapid ethnographic assessments prior to the study suggested that mobile clinics based in Mtubatuba taxi rank that provide hypertension and diabetes screening, and are supported by peer and sex worker outreach, would reach men who are mobile for work.

## Assignment of interventions: allocation

### Sequence generation {16a}

The allocation sequence will be generated using computer-generated random numbers using STATA. Randomisation will be done in a 1:1 ratio, stratified by setting (three groups) and using randomly permuted block size.

### Concealment mechanism {16b}

The generated allocation sequence will then be embedded within the data collection system (REDCap). At the time of enrolment, each participant will be given a unique randomisation identifier in the temporal order in which they are randomised within their setting. The trial arm allocation for this unique identifier will then be accessed in REDCap. The trial arm allocation will be hidden until the participant has received their unique randomisation identifier.

### Implementation {16c}

The allocation sequence will be written by the trial statistician and with the random seed chosen by a statistician who is otherwise uninvolved with the study. Participants will be enrolled by clinicians. After determining a participant’s eligibility, the clinician will log onto REDCap using their personal access credentials. REDCap will then prompt the clinician to confirm eligibility of the site, and that they want to randomise a new participant with the next randomisation ID for that site to the trial. Once confirmed, the participant is randomised by the clinician and assigned to a randomisation arm according to the next unique identifier on the allocation list. The randomisation allocation assigned will now be read-only and cannot be edited after this.

## Assignment of interventions: blinding

### Who will be blinded {17a}

Due to the nature of the trial, it is not possible to blind participants, study staff involved with participant care, or outcome assessors. Furthermore, data analysis will also be done unblinded due to the different schedules followed by participants depending on the trial arm to which they are allocated, which relates directly to how outcomes are ascertained and prohibits a blinded analysis.

### Procedure for unblinding if needed {17b}

Not applicable. This is an open-label study.

## Data collection and management

### Plans for assessment and collection of outcomes {18a}

#### Collection of clinical, sociodemographic, and behavioural data

Participants’ weight, height, and blood pressure will be measured at screening and at specified follow-up visits (Table 1), as injectable PrEP is potentially associated with weight gain and hypertension.

Sociodemographic, behavioural, risk, and mobility information will be collected through surveys at baseline and specified follow-up visits (Table 1). PrEP-related data, including details of the PrEP provided to and used by the participant, will be documented at each visit. During the “choice period” (9 to 18 months), participants will be asked at each visit to indicate their preferred PrEP intervention. For participants who choose to switch PrEP options, their reasons will be captured on a PrEP switch CRF. To evaluate PrEP adherence and ensure consistent counselling, a PrEP adherence CRF will be administered by a study team member at every visit. The PrEP adherence questionnaire will focus on the following:

- Self-reported PrEP use, including whether participants are currently taking PrEP, the number of days since their last PrEP tablet, and adherence surrounding condomless sex (e.g. the number of tablets taken the day before and after the sexual act)
- Any adverse clinical or social effects experienced as a result of PrEP use
- Reasons for pausing or discontinuing PrEP

### Collection of laboratory data

The following laboratory tests will be performed at screening and specified time points (Table 1) to confirm participant eligibility and the safety of product use.

- HIV testing: HIV will be confirmed at each in-person visit using the national HIV testing algorithm. Participants who test positive during the trial will be counselled and referred to local clinical centres for HIV care and treatment.
- Hepatitis B viral (HBV) testing: A blood sample will be collected at screening to test for hepatitis B infection using the site’s routine test for hepatitis B surface antigen.
- Creatinine clearance: Serum creatinine levels will be measured at screening for men aged 30 years and older. Subsequent testing will be conducted if clinically indicated.
- DBS: These samples will be collected and stored for later retrospective analysis of drug levels of drug levels and possibly viral load testing.
- Sexually transmitted infections (STIs): Testing for STIs, including gonorrhoea, chlamydia, and syphilis, will be performed based on local guidelines.

### Collection of social and economic science implementation data

To answer the secondary implementation objectives of reach, adoption, implementation, and maintenance using the RE-AIM implementation science framework (Table 2), a mixed-methods data collection approach will be adopted.

**Table 2:** The proposed RE-AIM evaluation

Domain definition	Implementation outcome	Data source
*R*each	*Client (user) level*: The extent to which mobile men at risk of HIV acquisition effectively take up PrEP measured as the patterns of uptake and persistence per arms and community experience	Rapid ethnographic assessments of each community and the surveys and process of engagement with services
*E*ffectiveness	*Client (user) level*: Increased effective use (adopt and adhere) of PrEP amongst mobile men. Measured as proportion of participants who are retained on PrEP and participant choice	Pragmatic randomised controlled trial (RCT)
*A*doption	*Client (user) level*: Acceptability (uptake when offered), attitudes towards, preferences/choices, and patterns of use amongst different groups of mobile men	Pragmatic RCT, surveys, qualitative interviews with service users & process of engagement with services, and exit interviews
*I*mplementation	*Provider (service) level*: Appropriateness, feasibility, practicability, and fidelity of CAB-LA in the three stages or initiation, continuation, and safe stopping with national HIV testing algorithms	Qualitative data from service providers, time motion studies, and HIV self-testing study
*M*aintenance	*Provider and health systems level*: Affordability and resources needed for scalability	Time motion studies and costing and modelling

Data collection methods will include the following:

#### Rapid ethnographic assessments

These assessments will be conducted at baseline, after 9 months, and at end line to provide implementation data on the reach of the intervention. The assessments will use a rapid ethnographic assessment (REA) methodology, a structured approach that combines a variety of qualitative methods in a sequential format, designed to move from broad contextual understanding to a more specific focus on the study topic. This approach allows for the collection of focused data that addresses both the whole community context as a whole and the specific behaviours and HIV-prevention choices of mobile men.

The REA methodology comprises a set of structured activities such as community entry, spiral walks, observations, individual conversations/interviews, natural group discussions, and focus-group discussions. This is aimed at gathering a comprehensive perspective on the area of study and the communities living there. The rapid ethnographic assessment approach is conducted within a defined 15-day period [51]. Community entry is conducted a few months prior to the commencement of data collection. The research teams engage with the Community Advisory Boards (CABs) overseeing the chosen research sites for guidance on appropriate community entry specific to the sites concerned [52, 53]. This is followed by seeking the relevant permissions from the local leadership, including traditional, political, or community leaders, depending on the area [54]. The data collection methodology is standardised across study areas, ensuring consistency and rigour in the process [39, 55].

#### In-depth qualitative interviews with service users

One-on-one interviews will be conducted with up to 10% of participants across the sites, based on the visit schedule. These participants will be purposively selected to represent the emerging patterns of risk and adherence behaviours identified from the quantitative data. These interviews will provide insights into the experience, motivations, and barriers that facilitate or obstruct adoption of each PrEP modality during the randomised phase and the rationale for adoption of each modality during the choice phase. In addition to exploring individual behaviour changes, the interviews will capture changes in the communities/places the participants stay to inform the trial about context-specific factors which may affect trial outcomes (e.g. loss of employment options in a location and changes in health care provision) and how participants move between PrEP choices.

#### Exit interviews (choices and trial participation)

Exit interviews will be conducted with all participants as they exit the trial to capture their experiences and reflections on the study. These interviews will focus on their reasons for trial participation, their PrEP choices, and their overall experiences with the intervention. Participants who seroconvert at any time during follow-up will also be invited to take part in an in-depth interview as described above. These interviews will explore key factors contributing to seroconversion, including details about condomless sex or PrEP adherence since their last negative result, as well as the number and characteristics of new sexual partners during the follow-up period.

#### Group discussions

These will be conducted with a subset of participants, typically comprising approximately eight participants per group. Group discussions will be led by staff trained in qualitative research methods, who are independent of the clinic team, to ensure objectivity and minimise potential biases. Participants will be purposefully selected based on specific criteria, while others will be invited on an ad hoc basis to ensure diverse perspectives. These group discussions will aim to explore participants’ experiences, perceptions, and insights on the trial, including their motivations, challenges, and changes in behavior related to PrEP use.

#### Interviews with service providers

Structured debriefs and one-on-one interviews with community and counselling staff will be conducted by a social scientist during the study. Typically, these interviews will capture conversations observed or initiated by staff with and between participants, nonparticipants, clinic staff, and community workers. The notes will be anonymous and documented using a standardised template. Special attention will be given to understanding how participants transition between ED and daily PrEP through conversations with a range of service providers, including mobilisers/recruiters. To support reflection and inform ongoing training and supervision, peer navigators and clinical staff will be trained to keep very brief notes of their daily activities. These notes will be used during debriefing sessions to discuss key observations and challenges. These interviews will take place once recruitment is closed; additionally, endline interviews are scheduled with service providers, stakeholders, and community members. Peer navigators will be interviewed, and natural group discussions will be facilitated with various stakeholder groups, such as community leaders, Community Advisory Board members, and local healthcare providers, to gather perspectives on the study's implementation and impact.

#### Collaborative problem-solving meetings

Meetings will be held with healthcare workers from all sites to allow for collaborative problem-solving. Implementation of the study will be discussed, and modifications made for improvement will be documented to inform the implementation analysis (implementation and adoption domains of the RE-AIM framework).

#### Quantitative survey

The survey will collect data on demographics (age, relationship status, education, income, location), mobility patterns, mental health, and social economic factors [food security, social cohesion (network of friends), and support systems]. Surveys at months 9 and 18 will additionally explore participants’ PrEP choices and the ratings of their experiences with PrEP using a Likert scale.

#### Health economics

Cost data will be collected using bottom-up and top-down costing, drawing on expenditure reports, resource use from CRFs, from the provider perspective. We will combine these with relevant, up-to-date information on public sector prices and salaries in either country. At the mid-point of implementation, data collectors will conduct a time-and-motion study with study staff involved in implementation at each site to estimate staff time and resources used on key activities. Trial resource use will be adapted to represent potential rollout in routine care.

### Plans to promote participant retention and complete follow-up {18b}

Measures to support PrEP persistence will include pre-dose counselling at each visit and standard retention efforts if the participant fails to report for a subsequent visit. All participants with a missed visit will be followed up in the following way:

- At day 7 post a missed visit, the participant will be called using the telephone number provided at the most recent visit. After three unsuccessful attempts on subsequent days, at different times, the study team will carry out a home visit.
- Participants will be strongly encouraged to come in irrespective of whether or not they need PrEP.
- For CAB-LA participants who fail to return within 7 days, a home visit will be conducted (if permission has been given and it is safe to do so). Once contacted, a study visit will be rescheduled as soon as possible. However, due to the risk associated with stopping CAB-LA without coverage for the tail period, participants in the CAB-LA arm will be followed up with further phone calls and a home visit (if permitted) following the 7-day period. If the participant indicates that they wish to withdraw from the study, then the withdrawal will be documented, with the reason for withdrawal noted if provided. If the participant is in the CAB-LA arm, the participant will be encouraged to take oral PrEP to cover the tail period and will receive counselling on safe sex.

Participants will not be considered lost to follow-up until the trial has ended, unless they have left the country with no plans to return and with no means to ascertain HIV status. The date of loss to follow-up will be the date of their last study visit.

### Data management {19}

Wherever possible, study data collected during clinic visits will be recorded directly into an electronic data capture system (REDCap). The data capture system will be programmed with quality control measures including range checks for quantitative variables. When direct data entry is not practical or is not available, the data will be recorded in a source document and will be transcribed into the electronic data capture system using double data entry within a reasonable timeframe. All electronic and nonelectronic documents and forms will be kept securely. Full details of data management procedures are provided in the study data management plan.

### Confidentiality {27}

All data will be encrypted and stored on password-protected servers, which have multifactor authentication activated to ensure secure access control. Each participant will be assigned a unique project identity number (ID) for capturing and storing their data. Personal identifiers will not be stored in the study data set, and all computers will be equipped with antivirus software and security passwords. Participant contact information will be kept separately from project documentation. While the study is in progress, study-related forms will be maintained in locked cabinets, with access restricted to authorised personnel only. Upon study conclusion, these documents will be digitised, indexed, and stored indefinitely on the central server. The original paper documents will be destroyed.

### Plans for collection, laboratory evaluation, and storage of biological specimens for genetic or molecular analysis in this trial/future use {33}

Samples will be collected according to the study laboratory manual and standard operating procedures. At each visit, DBS will be taken and stored for future analysis of drug levels and HIV viral load. The results of the dry blood spot analysis will not be given directly to the participants or clinic study staff but to the study team for the analysis. Furthermore, for individuals who acquire HIV during the study, blood will be taken for analysis including HIV viral load, resistance testing, drug levels, and storage for future use.

## Statistical methods

### Statistical methods for primary and secondary outcomes {20a}

The demographic, behavioural, and clinical characteristics of the two enrolled groups at baseline will be summarised, both overall and stratified by setting. All analyses will be done by intention to treat. No formal adjustment for multiplicity is planned for the co-primary effectiveness and implementation outcomes as the framework for effectiveness is superiority and that for implementation is exploratory.

For the primary user effectiveness outcome (persistence on PrEP during the randomised period), the odds ratio for persistence on PrEP comparing the two trial arms and a corresponding 95% confidence interval (CI), and *p*-value, will be generated using logistic regression, adjusting for setting (as randomisation is stratified by setting). The same approach will be used for binary secondary outcomes. For persistence during the full follow-up period, which is a multinomial outcome, multinomial logistic regression will be used, adjusted for setting.

For primary implementation outcomes, the odds ratio for uptake of PrEP comparing the two trial arms and a corresponding 95% CI, and *p*-value, will be generated using logistic regression, adjusting for setting (as randomisation is stratified by setting). Similarly, an odds ratio for the proportion who choose injectable compared to oral PrEP and a corresponding 95% CI, and *p*-value, will be generated using logistic regression, adjusting for setting.

A full statistical analysis plan will be finalised before database lock and will include sensitivity analyses to assess the impact of each element of the primary effectiveness outcome (which is a composite outcome) on study findings and to investigate a more flexible visit window for PrEP persistence.

### Interim analyses {21b}

No formal interim analyses are planned. Interim data on protocol adherence and safety will be reviewed by an Independent Data Monitoring Committee (IDMC), but no formal stopping guidelines are planned.

### Methods for additional analyses (e.g. subgroup analyses) {20b}

Subgroup analyses by study setting will be done to determine whether differences between trial arms are consistent across settings. These will be assessed by stratifying analyses by setting and testing for interaction.

### Methods in analysis to handle protocol non-adherence and any statistical methods to handle missing data {20c}

The primary analysis population will be all randomised participants, regardless of protocol adherence or use of PrEP, with the exception that participants who are discontinued from receiving their allocated PrEP for medical reasons will not be included in the primary user effectiveness outcome analyses. No per-protocol analyses are planned. Participants who are lost to follow-up will be included in primary user effectiveness outcome analyses, as they will be considered non-persistent on PrEP. If the proportion of participants with missing data is > 10%, then multiple imputation will be investigated; otherwise, complete case analysis only will be used.

### Qualitative data analysis

Data analysis for the rapid ethnographic assessments will be done manually using the framework analysis approach [56]. Manual analysis enables the three site teams to manage a range of data which consists of notes from observations, sketch maps, interview, and discussion group transcripts. Themes will be shared and discussed across the sites to arrive at a shared coding framework. Following the identification of themes, indexing (coding) and charting (cutting and pasting data according to thematic areas) will be done simultaneously at each site. Mapping (visual display of data) allows researchers to identify patterns, associations, and concepts, allowing descriptive and analytical memos to then be produced from these charts.

We will produce a short report to provide an overview for the wider study team on each community, and we will also aim to write a short feedback flyer to share with the communities based on the same short report. Once an initial report has been completed and summary findings shared with the participating communities, we will conduct a finer analysis of the data from the rapid ethnographic assessment, using a thematic content analysis—the approach we will also use for the qualitative data collected during the trial from interviews and group discussions.

For the qualitative data collected during the conduct of the trial from the IDIs and group discussions, we will again develop a shared coding framework, drawing from both the topic guides, learning from the rapid ethnographic assessments and new themes, which may come from the data collected. Analysis will be conducted by the site-specific research teams, and regular (electronic) meetings across the Ugandan and South African teams will support data interpretation. This will provide a more comprehensive understanding of men’s mobility and PrEP use across the trial groups and in the different settings/countries, as well as the acceptability and feasibility of the intervention. Where required to support the analysis, data sharing between sites will only involve the sharing of anonymised data through a secure data enclave.

### Health economics analysis

#### Cost analysis

We will analyse the incremental financial and economic costs of implementing either intervention over routine care for these populations from the perspective of the provider, the South African or Ugandan government. Standard of care approaches to retaining people in care will be identified from routine government services for HIV in each recruiting country. Total and average cost per person retained at 3 and 6 months will be estimated from the perspective of the provider and the public healthcare system in each country. Additionally, we will estimate PrEP coverage during periods of sexual activity. We will calculate annualised capital and recurrent costs at facility and any other relevant implementation level. Non-annualized annual economic costs will be used to inform the budget impact for each country and inform governments and other relevant funders of the intervention’s affordability.

### Cost-effectiveness analysis

We will combine results regarding the incremental cost from above and HIV synthesis model outputs regarding the incremental number of infections averted, life-years saved, and disability-adjusted life-years (DALYs) averted by each strategy (oral or injectable PrEP) over baseline (no scale-up of PrEP) to calculate the incremental cost-effectiveness of each intervention, as well as the strategy of offering either intervention. The HIV synthesis model will be fitted to epidemiological data to represent the current HIV epidemics in South Africa and Uganda and be parameterised and structured to account for important behavioural heterogeneity, including how behaviours and PrEP choices vary among cohort participants and within persons over time. The synthesis model will be used to calculate costs over the long term, as well as life-years lost and DALYs, with costs and effectiveness calculated over a sufficiently long period to represent the difference in the main impacts between strategies but at least over 20 years.

### Plans to give access to the full protocol, participant-level data, and statistical code {31c}

The full protocol, participant-level dataset, and statistical code will all be available upon request. Additionally, a metadata-only record will be created to catalogue the resources, including qualitative and quantitative data, codebooks, data dictionaries, algorithms, scripts, and research instruments such as questionnaires and interview guides. These resources will be archived in the LSHTM Data Compass (https://datacompass.lshtm.ac.uk), a curated digital repository designed to host materials that support the verification and reproduction of research findings. The study protocol is registered on ClinicalTrials.gov (NCT06133686) and the Pan-African Clinical Trials Registry (PACTR202409632006463).

## Oversight and monitoring

### Composition of the coordinating centre and trial steering committee {5d}

#### The Trial Management Group

The Trial Management Group (TMG) comprises the chief investigator, coordinating investigator, all investigators, representatives of ViiV Healthcare (investigational product providers), key trial site staff, and members of the MRC/UVRI and LSHTM Uganda Research Unit Trial Coordinating Centre. The TMG is responsible for the day-to-day running and management of the trial.

### The Trial Management Team

The Trial Management Team (TMT) comprises the chief investigator, coordinating investigator, trial statistician, other investigators (clinical and non-clinical) with operational coordination responsibilities, and members of the MRC/UVRI and LSHTM Uganda Research Unit Trial Coordinating Centre. The TMT is responsible for the overall management and coordination of the trial, including the following: protocol development, data management, laboratory coordination, regulatory and ethical compliance, procurement and supplies, maintaining the Trial Master File (TMF) and ensuring compliance with good clinical practice (GCP), development of the statistical analytical plan (SAP), and preparing reports for the IDMC.

### The Trial Safety Group

The Trial Safety Group (TSG) comprises medically qualified members of the TMT who are not directly involved in the clinical management of trial participants. The TSG is responsible for ensuring participant safety throughout the trial; evaluating all SAE reports and providing expert advice on further investigation and management; offering guidance on the clinical management of events affecting trial participants; reviewing updated safety data, including changes to the Summary of Product Characteristics (SmPC) for PrEP, and emergent safety reports from product providers; and determining the implications of new safety information on the trial.

### The Trial Steering Committee (TSC)

The TSC comprises key study investigators, including representatives from each participating trial site, as well as independent researchers with expertise in HIV prevention and PrEP in sub-Saharan Africa. The TSC acts as an oversight body delegated by the sponsor, providing strategic guidance and ensuring the trial’s conduct aligns with its objectives. It provides advice through its independent chair to the Trial Management Team (TMT) and Trial Management Group (TMG) on all aspects of the trial. Members formally register their agreement to join the committee.

### The independent data monitoring committee (IDMC) {21a}

The IDMC is composed of experts in HIV prevention both internationally and in Africa. The main responsibility of the IDMC is to safeguard the interests of the trial participants and monitor safety and overall trial conduct. The committee will review accumulating trial data and make recommendations to the TSC and the sponsor regarding trial modification, continuation, or termination. The IDMC members will formally sign an assent to join the IDMC and declare any competing interests. Further information regarding IDMC-specific responsibilities, terms of reference, meetings, and communication is detailed in the IDMC charter.

### Adverse event reporting and harms {22}

Information on AEs will be collected through an open question about health at study visits. Study staff will record the diagnosis or the symptoms if a diagnosis is not apparent, the date of onset, and the date of resolution if appropriate. If the event is ongoing, it may be appropriate to conduct a symptom-directed examination. Events will be graded according to the Division of AIDS (DAIDS) Table for Grading the Severity of Adult and Paediatric AEs version 2.1, July 2017 [57]. The relationship to CAB-LA or oral PrEP will be determined by the investigator. All this information will be recorded on the appropriate CRF. If the participant is unable to attend a safety follow-up visit for personal reasons, the interview may be conducted on the telephone or at a home visit if the participant is agreeable. Study staff will make every effort to follow AEs to resolution or stabilisation. After the trial has closed and the database has been locked, additional information regarding SAEs that comes to the attention of the study staff will be reported by email to the chief investigator and study sponsor.

### Frequency and plans for auditing trial conduct {23}

Risk-based monitoring will be implemented and conducted by the study sponsor-designated monitors. Both onsite monitoring that involves an in-person evaluation of study conduct at the site and remote monitoring that involves relying on the site to provide the required information while using digital platforms will be undertaken. Site visits will occur starting with a site qualification visit conducted prior to the study Site Initiation Visit (SIV). The SIV will be conducted prior to study start after all requisite ethical and regulatory approvals have been obtained. At each site, six interim monitoring visits will take place at 3 to 6 monthly intervals, with a combination of onsite and remote interim monitoring visits in keeping with the monitoring plan. Monitoring will also be based on the site risk review, upon which a remote or site visit may be conducted (triggered site monitoring visit). A close-out monitoring visit will occur after the last participant’s last visit, data cleaning, and investigational medicinal product inventory have been completed. Sites will be subject to audits should these be required by the sponsor. Ethics and regulatory authority inspections will also be permitted. Appropriate consent for monitoring, audits, and inspections will be obtained from the study participants.

### Plans for communicating important protocol amendments to relevant parties (e.g. trial participants and ethical committees) {25}

Any changes to the protocol will be submitted to relevant ethics committees and regulatory authorities for review and approval. Where applicable, changes will be shared with trial participants through one-on-one interactions with trial staff during follow-up visits. Amendments that result in changes to consent documents will require the re-consenting of trial participants. Relevant updates will be made to the trial registry in case of protocol amendments.

## Dissemination plans {31a}

A whole or part of this trial’s results will be communicated, orally presented, and/or published in appropriate scientific journals and at both local and international academic conferences. Full anonymity of participants’ details will be maintained throughout. Participants can request a copy of the journal article that contains the results of the trial from the investigators once it has been published. Results will be disseminated to the participants in the study during a formal dissemination event that accommodates a question-and-answer session to support full understanding of the study results by the participants.

## Discussion

This multicountry, mixed-methods study seeks to evaluate the effectiveness and acceptability of PrEP among men who are mobile for work in sub-Saharan Africa, a group at heightened risk for HIV acquisition. Through a hybrid implementation-effectiveness RCT, we will assess both ED oral PrEP and CAB-LA as flexible prevention options designed specifically to meet the needs of mobile men. The findings from this RCT, complemented by qualitative data, will help inform future PrEP delivery strategies tailored to this high-risk population. The study will gather critical insights through IDIs, group discussions, and qualitative surveys to understand how mobile men approach PrEP use. These qualitative methods will uncover practical challenges, preferences, and barriers, helping to shape PrEP interventions that align with the unique lifestyles and mobility patterns of men engaged in work-related travel.

In South Africa, the trial will be conducted through mobile clinics, made possible by a paperless study system. A rapid ethnographic assessment provided key insights into each participating community, allowing the research team to anticipate and address potential operational challenges. This proactive planning ensured a smooth trial launch.

The use of the RE-AIM framework in this trial provides a structured, comprehensive approach to evaluating the real-world implementation of both oral and injectable PrEP for mobile men in sub-Saharan Africa. By extending beyond clinical efficacy, the RE-AIM framework enables a real-world assessment of PrEP’s performance, identifying facilitators and barriers to access within mobile clinic settings. These insights will inform targeted adjustments in PrEP messaging, education, and delivery, ensuring they are tailored to the unique needs of mobile men. The RE-AIM framework enhances the study’s capacity to inform scalable and impactful HIV prevention strategies for this high-risk group.

## Trial status

Recruitment commenced on 20 August 2024 at the Desmond Tutu HIV Foundation (DTHF) and on 10 September 2024 at the Africa Health Research Institute (AHRI) in South Africa. In Uganda, recruitment commenced on 16 October 2024. Recruitment in both countries concluded on 8 November 2024. The manuscript is submitted shortly after completion of trial recruitment; it is not submitted earlier due to the trial team focusing efforts on ensuring that the necessary ethical and regulatory approvals were in place to enable rapid trial initiation and participant enrolment. Participant follow-up and data collection are ongoing and expected to conclude by April 2026. The trial is being conducted under protocol version 3.0, dated 9 April 2024.

## Data Availability

The original dataset will only be available to researchers directly involved in the study, and will be kept on secure, access-restricted servers. Anonymised data will, upon publication of the study, be made available upon reasonable request to the principal investigator.

## Abbreviations

AGYW: Adolescent girls and young women
AHRI: Africa Health Research Institute
AIDS: Acquired immunodeficiency syndrome
ART: Antiretroviral therapy
BCM: Buffalo City Metro
BMI: Body mass index
BREC: Biomedical Research Ethics Committee
CAB-LA: Cabotegravir long-acting
CI: Confidence interval
CRF: Case report form
DAIDS: Division of AIDS
DBS: Dry blood spot
DTHF: Desmond Tutu Health Foundation
ED: Event-driven
EDCTP: European and Developing Countries Clinical Trials Partnership
EU: European Union
FTC: Emtricitabine
GCP: Good clinical practice
HBV: Hepatitis B virus
HIV: Human immunodeficiency virus
HPTN: HIV Prevention Trials Network
HREC: Human Research Ethics Committee
IDMC: Independent Data Monitoring Committee
IMP: Investigational medicinal product
KCL: King’s College London
LSHTM: London School of Hygiene & Tropical Medicine
MRC: Medical Research Council
RCT: Randomised controlled trial
SA: South Africa
SAE: Serious Adverse Event
SAP: Statistical Analysis Plan
SAPHRA: South Africa Health Products Regulatory Authority
SIV: Site Initiation Visit
SMS: Short message service
SQV: Site Qualification Visit
STD: Sexually Transmitted Disease
TASP: Treatment as prevention
TB: Tuberculosis
TDF: Tenofovir disoproxil fumarate
TFV: Tenofovir
TMF: Trial master file
TMG: Trial management group
TMT: Trial management team
TSC: Trial steering committee
UNAIDS: Joint United Nations Programme on HIV/AIDS
USA: United States of America
UVRI: Uganda Virus Research Institute
WHO: World Health Organisation
